# Regulated cell death in fungi from a comparative immunology perspective

**DOI:** 10.1038/s41418-025-01570-z

**Published:** 2025-09-03

**Authors:** Asen Daskalov

**Affiliations:** 1 State Key Laboratory for Managing Biotic and Chemical Treats to the Quality and Safety of Agro-products, Institute of Plant Protection and Microbiology, Zhejiang Academy of Agricultural Sciences, Hangzhou, China; 2 Univ. Bordeaux, CNRS, ImmunoConcEpT, UMR 5164, F-33000 Bordeaux, France

**Keywords:** Microbiology, Immunology

## Abstract

The death of fungal cells has been studied in a variety of contexts including responses to antifungal drugs, during fungal developmental processes, in response to bacterial or mycoviral fungal pathogens, and during non-self-recognition between distinct strains of the same species (allorecognition). Some of the genetic determinants and molecular mechanisms of fungal cell death processes are now beginning to be understood in detail. Recent advances have uncovered fungal cell death machinery that shares ancestry with key actors of immune cell death in other eukaryotic and prokaryotic taxa. Transkingdom evolutionary links include fungal molecular sensors such as NOD-like receptors and signaling domains related to the TIR (Toll/interleukin-1 receptor) family, which are a staple of immunity throughout the tree of life. Moreover, cell death executioner proteins homologous to the pore-forming proteins that mediate mammalian *necroptosis* and *pyroptosis* are also abundant and widespread in fungi, particularly in Ascomycota. These findings prompt us to speculate on the possible origins of fungal cell death and to reconsider fungal innate immunity beyond allorecognition. This review discusses historical landmarks and major recent discoveries regarding the regulation of cell death processes in fungi through the lens of immunity.

## Facts

- Fungal regulated cell death involves molecular machinery that is distantly related to cell death pathways in other eukaryotic and prokaryotic taxa.
- Many fungal cell death determinants have been identified through studies of heterokaryon incompatibility (HI), a prophylactic cell death process occurring during conspecific non-self-recognition.
- Fungal genomes encode a diverse repertoire of NOD-like receptors, some of which are involved in cell death and non-self-recognition.
- Fungal amyloid-signaling and HeLo/HELL membrane-targeting domains establish an evolutionary link with *necroptosis* and RCD in plants.
- Fungal gasdermins (fGSDMs) and their cognate proteolytic regulators draw evolutionary parallels with *pyroptosis*.

## Open questions

- What molecular cues are recognized by each of the numerous fungal NOD-like receptors?
- What is the full range of diversity of defense-related cell death and immunity mechanisms that exist across fungal species?
- What is the level of interconnectedness of the cell death pathways constituting fungal immunity systems?
- Are distinct fungal cell death pathways associated with specific morphological hallmarks?

*Regulated cell death* (RCD) refers to cellular suicide in both multicellular and unicellular organisms. RCD relies on genetic determinants and signaling pathways, providing points of genetic and pharmacological control and with stimulus- or context-dependent specificity to the process [1, 2]. In animals, a variety of RCD pathways participate in the maintenance of homeostasis by eliminating damaged, malfunctioning and potentially harmful cells from the body [3, 4]. Here we refer to fungal cell death as RCD, as defined by the functional and sequential interactions between the involved molecular players and associated with specific morphological and biochemical hallmarks of cell disintegration [4].

## Regulated cell death is a shared defense strategy between eukaryotic and prokaryotic taxa

Comparisons between immune systems of distant taxa provide an opportunity to understand the underlying, universal principles of immunity and identify major taxa-specific distinctions. *Regulated cell death* is a shared defense strategy between eukaryotic [5] and prokaryotic taxa [6]. Simply put, the strategy consists of eliminating infected cells to limit or prevent the multiplication and spread of a pathogen. In the case of single-cell prokaryotes including many bacteria and archaea, the benefits of cell death occur at the population level, notably preventing the propagation of phages [7, 8]. In plants, the *hypersensitive cell death* *response* (HR) activates at the point of penetration by a plant pathogen (e.g. bacteria, virus, fungi) and helps contain the infection providing resistance to the pathogen invasion, playing a key role in the plant immune system [9–11].

Multicellular animals (Metazoa) are a sister clade of Fungi, and together form the eukaryotic supergroup of Opisthokonta [12, 13]. In animals and particularly in mammals, many distinct RCD pathways have been extensively characterized, since the introduction of the term *apoptosis* in 1972 [14]. Apoptotic cell death plays an essential role in animal development, homeostasis and immunity [15–17]. Cells undergoing apoptosis are dismantled in an orderly manner, with minimal dispersal of cellular content [18], while other mammalian RCD pathways, like *necroptosis* [19–21] and *pyroptosis* [22–24], are lytic and highly inflammatory. *Necroptosis*, *pyroptosis* and *ferroptosis* are three of the best understood pathways and also core cell death programs of mammalian immunity [4, 25–27]. Remarkably, exploration of fungal immune defense-related RCD has yielded thus far, several findings establishing evolutionary ties between some fungal RCD-controlling proteins and crucial players in mammalian *pyroptosis* and *necroptosis*.

This review describes current fungal RCD research with an emphasis on recent discoveries that tie fungal cell death-controlling proteins to immune systems across the tree of life. A particular focus is placed on comparisons within Opisthokonta, where the metazoan pathways are characterized in great detail. Comparisons are further extended to bacterial and plant immunity genes, underscoring the ancient evolutionary origins of some cell death programs. By considering the historical context, I discuss our evolving understanding of fungal RCD and propose that a comparative immunology framework offers an ambitious, productive, and systematizing approach for future research on fungal regulated cell death.

## Heterokaryon Incompatibility is a fungal prophylactic cell death response that restricts mycoparasitism

Cell death in fungi has been investigated in the context of fungal pathogenesis by examining fungal responses to antifungal drugs and other antimicrobial compounds [28, 29]. Conversely, fungal RCD has also been investigated in fungi infected by fungal viruses (mycoviruses) [30] and during fungal–bacterial interactions [31–33]. In addition, RCD plays an important role in various physiological processes in fungi. These include the differentiation of specialized cellular structures for reproduction (*protoperithecia*) [34], for host invasion purposes (*appressoria*) [35–37], and during sexual sporulation in both filamentous fungi [38, 39] and yeast [40].

**Fig. 1:**
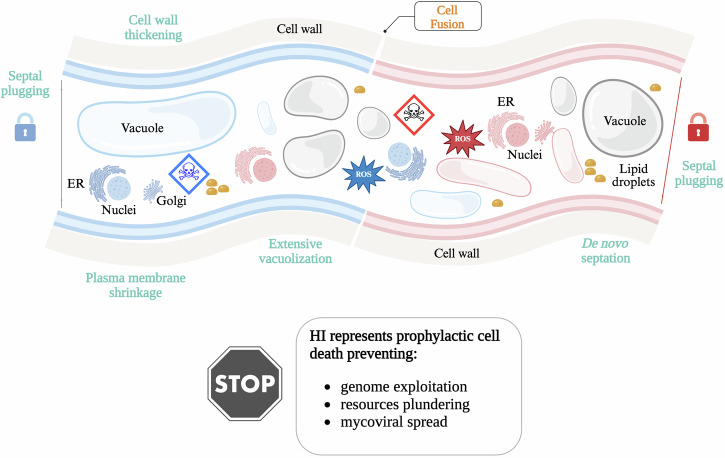
Heterokaryon incompatibility (HI) represents a prophylactic cell death reaction in fungi, limiting exchanges between genetically distinct fungal colonies. Regulated cell death (RCD) occurs post-anastomosis – a cellular fusion process. The illustration shows a blue hyphal cell from one colony fusing with a red hyphal cell from a genetically distinct, incompatible individual. Generally, the newly formed heterokaryon is rapidly isolated from both colonies – symbolized by keylocks – by plugging the pores that connect adjacent cells in the hyphae. The HI cell death process is characterized by massive vacuolization, production of reactive oxygen species (ROS), and the formation of lipid droplets. Additionally, cell wall thickening and de novo septation occur—although these features are not depicted in the cartoon. Subsequently, the HI cell death culminates in plasma membrane shrinkage and rupture, releasing the cytoplasm of the dead fusion cells.

A major part of our current understanding of fungal RCD stems from historical studies of *conspecific non-self-discrimination* (also known as *allorecognition*), which occurs during somatic cell fusion between genetically distinct fungal colonies [41–43]. Notably, this phenomenon has been compared to graft rejection in mammals, where the immune system recognizes and rejects non-self tissues [44, 45]. Allorecognition has also been studied in basal metazoans (sponges and corals) [46, 47], social amoebae [48], and bacteria [49]. In fungal species like *Neurospora crassa*, non-self recognition and discrimination between distinct strains can occur at a distance (pre-contact) [50], at the point of contact before cellular fusion [51], or after somatic fusion of two strains. Incompatibility between strains may arise independently at any of these three stages due to genetic factors. In the first two cases, which occur prior to cellular fusion, the strains remain viable. However, if two genetically incompatible strains undergo somatic cell fusion (*anastomosis*) to form a heterokaryon, a lytic cell death process is triggered within the heterokaryon [42, 52, 53]. This abortive *anastomosis* event is known as *heterokaryon incompatibility* (HI) and is characterized by extensive vacuolization, plasma membrane shrinkage, elevated reactive oxygen species (ROS) levels, and lipid droplet production [52, 54] (Fig. 1). The heterokaryotic fusion cells undergoing HI are sequestered from the rest of the colony by plugging the large channels through which cytoplasm and organelles normally flow [55]. New septa (cell membrane partitions) form de novo in the dying cells before lysis completes and the cells disintegrate, severing the connection between incompatible strains [54].

### *het* genes control regulated cell death during heterokaryon incompatibility

**Fig. 2:**
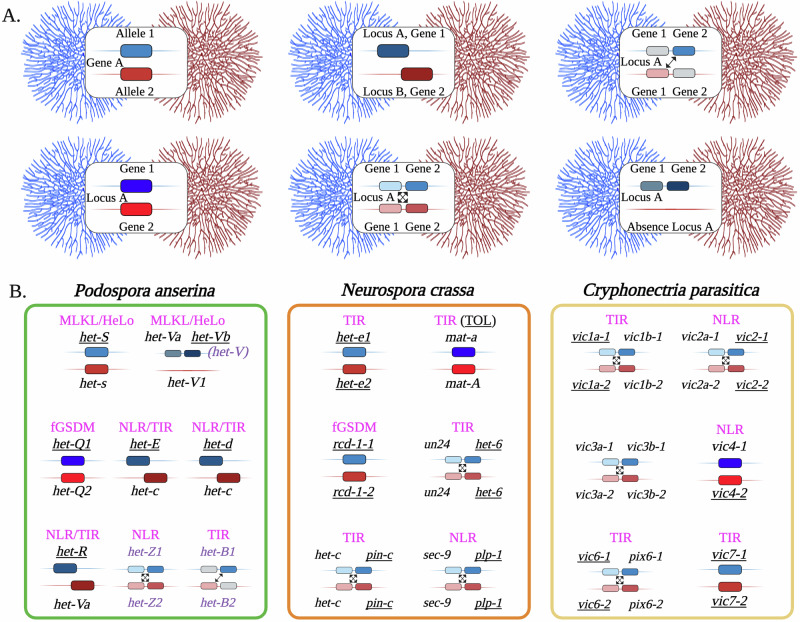
Genetics of heterokaryon incompatibility (HI) in filamentous fungi. **A** HI is triggered by genes termed *het* or *vic* (vegetative incompatibility), which define independent ‘incompatibility systems’ when sharing the cytoplasm of a heterokaryotic cell originating from the fusion of genetically incompatible strains, shown as blue and red colonies. HI systems can be formed by the antagonistic alleles of the same gene or by different genes. In the latter case, the RCD-inducing genes may be at the same locus in the genomes of the incompatible strains (HI system between idiomorphs) or consist of two antagonistic genes situated at different loci. Some HI systems rely on tightly linked genes, in which the RCD-inducing interactions among these *het* gene products can be symmetrical or asymmetrical (indicated by arrows). An HI system (V/V1) has also been described in *P. anserina*, in which the absence of a specific locus in one strain can lead to RCD in heterokaryons formed with another strain that possesses this locus. **B** Molecularly characterized HI systems in three model ascomycete species used for investigating fungal allorecognition and cell death. The characterization of almost all HI systems in these species reveals evolutionary parallels with genes and protein domains that regulate immunity and cell death throughout the tree of life, as indicated by the fuchsia highlighting each HI system representation. Genes establishing the evolutionary link are underscored. Genetic loci containing two *het* genes (*het-V*, *het-Z*, and *het-B* in *P. anserina*) are highlighted in purple. The molecular characterization of *het*/*vic* genes is presently exhaustive for both *P. anserina* and *C. parasitica*; however, several genetically mapped *het* loci in *N. crassa* remain to be explored and characterized molecularly (not shown in the figure).

Exploration of the cell death mechanisms occurring during HI has led to the discovery of the genetic bases of fungal RCD. Early classical genetic studies identified several genetic loci responsible for cell death during somatic cell fusion between incompatible fungal strains [41]. In most species – including *Neurospora crassa*, *Podospora anserina*, and *Aspergillus fumigatus* – the genes are named *het* (short for heterokaryon incompatibility), whereas in *Cryphonectria parasitica*, they are called *vic* genes (for vegetative incompatibility). The genetic interactions between *het* genes define *heterokaryon incompatibility (HI) systems*. Each HI system is independent of the others in its ability to trigger incompatibility, and their total number per species typically does not exceed a dozen – often fewer. Such HI systems can arise from interactions between allelic variants of a single gene or between unrelated genes, which may be located at distant loci or, more commonly, represent two tightly linked genes [41, 56] (Fig. 2).

### Heterokaryon incompatibility limits the spread of fungal viruses (*mycoviruses*)

HI limits cytoplasmic exchanges between incompatible fungal strains, thereby preventing the horizontal transmission of mycoviruses and other genetic elements [57–62]. Mycoviruses can impair infected strains by reducing their fitness and virulence, resulting in *hypovirulent* fungal strains [63]. Therefore, some researchers are harnessing the features of HI systems, and mycoviruses, as biocontrol strategies to combat plant-pathogenic fungi [63, 64]. HI has been found to limit the spread of viral and other selfish cytoplasmic elements (such as senescence plasmids) in *Aspergillus niger* [65], *Neurospora crassa* [66], *Podospora anserina* [67], and *Cryphonectria parasitica* [57].

In effort to develop anti-fungal strategies against chestnut blight disease, the relationship between HI systems and mycoviral spread has been most thoroughly explored in the pathogenic fungus *Cryphonectria parasitica*. Studies of *C. parasitica* HI systems have led to the molecular characterization of six distinct incompatibility systems [68, 69]. The efficiency of *Cryphonectria* HI systems in restricting mycoviral transmission – defined as the infection of a virus-free recipient strain through somatic cellular fusion with a virus-infected donor – varied among the six *vic* loci [70]. Heteroallelism at each of five *Cryphonectria* HI systems individually restricted mycoviral transmission between strains, with some *vic* loci reducing transmission rates by 80%. In contrast, the remaining one of the six HI systems had no impact on viral transmission under the tested conditions [70]. When tested independently, gene disruptions that inactivate any one of these five HI-controlling systems – and thereby abolish prophylactic HI cell death – result in increased viral transmission between strains in both laboratory [57, 68, 69] and natural environments [58]. By simultaneously shutting down four of the five virus-restricting HI systems, Zhang and Nuss successfully generated super mycovirus donor strains capable of transmitting the virulence-attenuating CHV1/EP713 mycovirus (*Cryphonectria hypovirus 1*, isolated from strain EP713) to a broad range of wild isolates [57].

For some *Cryphonectria* HI systems composed of two tightly linked genes (Fig. 2), disruption of a single gene can increase mycoviral transmission without completely abolishing the cell death event between the incompatible strains [57, 69]. These findings, along with the discovery that mycoviral transmission frequencies can depend on the mycoviral donor-recipient status of a strain [70], suggest that some HI systems may be under selective pressure to specifically block mycoviral infection. As expected for virus-host interactions, some mycoviruses have also evolved mechanisms to suppress HI systems, facilitating their horizontal intraspecies transmission [71]. Although the precise molecular details of viral counter-defense mechanisms remain unclear, some viruses appear to downregulate the transcription of fungal allorecognition genes [71]. An important recent study shows that plant hosts can also suppress HI systems in the pathogenic fungus *Sclerotinia sclerotiorum*, thereby facilitating mycoviral transmission and reducing the virulence of this fungal pathogen [72].

### Heterokaryon incompatibility as a defense against resource plundering and cheater exploitation

HI cell death can also protect fungal colonies from other hazards, such as resource plundering [73] and cheater genotypes that exploit the genomes of conspecific non-self-strains [74, 75]. These detrimental situations arise because anastomosis is a key aspect of fungal lifestyles, and many fungal species are characterized by a multinucleate, syncytial organization. An example of resource plundering has been described between strains of *N. crassa*, which develop specialized ‘paternal’ cells (*conidia*) and ‘maternal’ reproductive structures (*protoperithecia*) to undergo the sexual cycle. Debets et al. reported that some paternal nuclei gain access to the maternal colony when conidia from the paternal line vegetatively fuse with the maternal protoperithecia-developing strain, thereby exploiting the resources that the maternal colony dedicates to sexual differentiation and sporulation [73]. The success of this ‘resource plundering’ strategy is significantly reduced by allorecognition-mediated cell death between the two strains [73]. Notably, the mating type locus defines one of the somatic HI systems in *N. crassa* [76, 77]. A similar context and logic apply to *N. crassa* nuclei carrying mutations that reduce investment in somatic functions such as conidiation, while providing reproductive advantages (‘cheaters’) compared to non-cheaters [75]. HI reduces heterokaryon formation with such cheater genotypes, further exemplifying the benefits of this prophylactic cell death as a potent defense strategy against parasitism.

### Evolutionary signatures of *het* genes highlight the adaptive role of heterokaryon incompatibility in nature

The importance of HI in nature is reflected in the evolutionary hallmarks of *het* genes. Notably, *het* genes are generally highly polymorphic, and the alleles of many appear to be under *balancing selection* [78]. As a result, *het* alleles within a given HI system tend to be maintained at nearly equal frequencies in wild populations [79–82]. This pattern is thought to arise from the interdependency between the adaptive value of each *het* allele and its frequency. That is, strains harboring rarer alleles can recognize and reject a wider range of incompatible partners, conferring a selective advantage over more common variants. Over time, these initially rare alleles increase in frequency, displacing previously common alleles, which in turn become rarer [78]. Some heterokaryon incompatibility (HI) systems even transcend species boundaries, leading to *trans-species polymorphism* – where the same alleles are shared by closely related species rather than strictly sorted along species lines [79, 83]. Both balancing selection and trans-species polymorphism have been associated with immunity-related genes in animals and plants [84–87]. These evolutionary hallmarks underscore the significant benefits *het* genes confer in nature, enabling fungi to maintain robust defenses against conspecific non-self and its associated hazards.

## Heterokaryon Incompatibility reveals a treasure trove of genes inducing fungal RCD

**Fig. 3:**
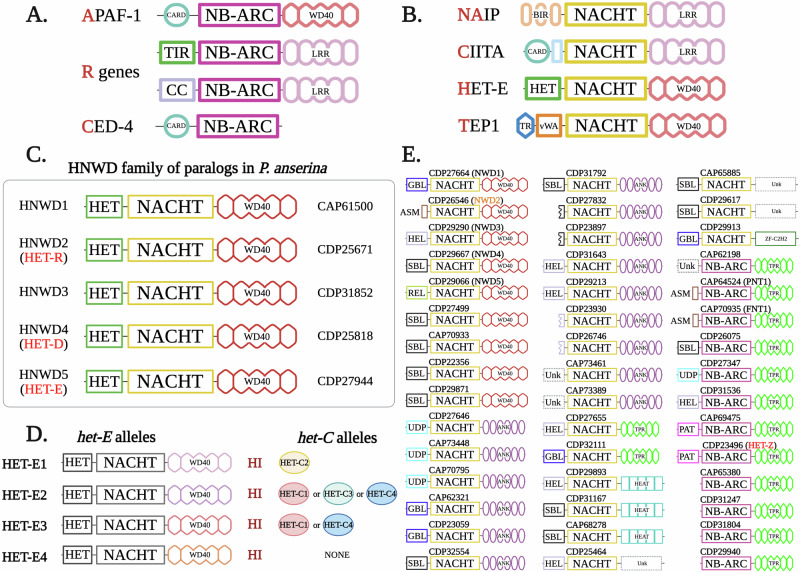
NOD-like receptors (NLRs) and NLR-like proteins form a transkingdom gene superfamily with conserved roles in immunity and regulated cell death. NLRs are defined by their central nucleotide-binding (NB) and oligomerization (NOD) domains. NODs generally belong to either the NB-ARC family or the NACHT family. **A** Founding members of the NB-ARC family include mammalian APAF-1 (Apoptotic protease activating factor 1), its homolog CED-4 (Cell death protein 4) from the roundworm *Caenorhabditis elegans*, and plant R (resistance) proteins. **B** Founding members of the NACHT family include three animal proteins – NAIP (Neuronal apoptosis inhibitory protein), CIITA (class II, major histocompatibility complex transactivator), and TEP1 (Telomerase associated protein 1) – and HET-E (heterokaryon gene), which is a protein encoded in the genome of the mold *Podospora anserina*. **C** NOD-like receptors encoded in the genome of the ascomycete *Podospora anserina*, a fungal model organism for the study of regulated cell death and NLR biology. The *hnwd* (HET-NACHT-WD40) gene family comprises five paralogs, three of which (*het-r*, *het-d*, and *het-e*) control heterokaryon incompatibility (HI). The family is characterized by the presence of a HET domain—likely related to the TIR (Toll/interleukin-1 receptor/resistance protein) domain—at the N-termini of its five members. The WD40 pseudo-repeats of the *hnwd* genes exhibit strong internal conservation and are proposed to evolve in a concerted manner. Repeats can be exchanged between alleles of the same gene or between different members of the gene family. **D** Interallelic genetic interactions define the *het-e*/*het-c* HI system in *P. anserina*. Different alleles of *het-e* (e.g., *het-e1*, *het-e2*, *het-e3*), which differ almost exclusively in the number and composition of their WD40 repeats, trigger HI in the presence of distinct alleles of the *het-c* gene. The *het-c* gene encodes a glycolipid transfer protein that is broadly conserved across fungi. Some functionally characterized *het-e* alleles (e.g., *het-e4*) appear inactive in heterokaryon incompatibility (HI) and typically carry a low number of WD40 repeats, while others (e.g., *het-e2*) can induce RCD in the presence of different *het-c* alleles. Experimental data have demonstrated that the specificity of recognition between *het-e* and *het-c* allelic variants depends on the WD40 domains of the NLRs, and the cell-death reaction relies on a functional HET/TIR domain. The *het-D* paralog is involved in a very similar HI system with *het-c*. **E** Cartoon representation of 45 *P. anserina* NOD-like receptors. Genes directly involved in heterokaryon incompatibility (HI) are highlighted in red, and those indirectly involved are shown in orange. *Abbreviations*: HEL (HeLo-like in fungal proteins), PAT (Patatin-like phospholipase), SBL (SesB-like lipase), GBL (Goodbye-like domain), UDP (UDP-glucose phosphorylase domain), HET (*Het*erokaryon determinants domain), ASM (Amyloid Signaling Motif), CARD (Caspase recruitment domain), BIR (Baculovirus IAP repeat), TR (Telomerase, Ro and Vault or TROVE domain), vWA (von Willebrand A domain), TPR (Tetratricopeptide repeat), ANK (Ankyrin repeat), and Unk (unknown).

To date, molecular characterization of *het* genes has led to two striking observations: (i) *het* genes are evolutionarily related to innate immune genes that regulate RCD in both prokaryotic and eukaryotic organisms, including mammals; and (ii) they belong to large and widespread gene families. The gene families containing the most *het* genes often comprise dozens or even hundreds of genes per genome and their numbers can vary greatly between species. One large gene family, to which many *het* genes belong, encodes fungal NOD-like receptors (NLRs) [88] (Fig. 3). NOD-like receptors are ancient intracellular sensors that couple immune detection to regulated cell death across animals, plants, and prokaryotes. Frequently, *het* genes also encode proteins containing evolutionary distant TIR (Toll/interleukin-1 receptor) domains [83], which are found on immunity proteins in animals, plants, and bacteria [89]. In fungi, such TIR-like domains are also known as HET domains – a name derived from their frequent association with identified *het* genes [83, 90]. Other *het* genes are members of the fungal gasdermin (fGSDM) family [91, 92], distant evolutionary relatives of mammalian gasdermins, executioner proteins of *pyroptosis* [93]. A smaller group of characterized *het* genes belongs to a large gene family encoding proteins with membrane-targeting coiled-coil domains [94, 95], which are related to the *necroptosis* executioner protein MLKL [96] (Fig. 2).

### Fungal *het* genes are part of broad gene families with proposed roles in RCD and immunity

Importantly, the vast majority of fungal NLRs, fGSDMs, and TIR/HET domain-encoding genes are not involved in allorecognition, indicating broader roles beyond incompatibility responses. Consistent with this, genetic mapping in the few model species used to study HI has consistently identified only six to a dozen *het* loci per species [41, 43]. To clarify the relationship between *het* genes and other members of their gene families, it has been proposed that HI-defining loci were co-opted from large pre-existing gene pools primarily involved in *xenorecognition* (discrimination between species) and innate immunity [97–99]. This hypothesis is further supported by uncovered functional similarities between gene members across kingdoms – such as fGSDMs, NLRs, and TIR-containing receptors from other taxa – which include known regulators of immune cell death in the context of xenorecognition [97]. Aligned with this perspective, although *het* genes govern prophylactic cell death in heterokaryotic chimeras, most members in these gene families (NLRs, fGSDMs, TIR/HET domain-encoding genes) are likely involved in the regulation of RCD in other contexts, including *direct* defense against pathogens and antagonists that target fungi. Together, the *het* genes and the (non-*het*) members of the gene families would form the allorecognition and xenorecognition molecular mediators, respectively, of the fungal immune system [97–99].

### NOD-like receptors and the STAND superfamily: conserved intracellular mediators of immunity and cell death

Signal-transducing ATPases with numerous domains (STANDs) are a superfamily of intracellular receptors conserved in prokaryotes and eukaryotes, associated with non-self discrimination, immunity, and regulated cell death [100]. The NTPase module binds and hydrolyzes nucleotides, playing an important role in the oligomerization of the sensors, which can be activated by endogenous or exogenous signals [101]. Two of the major families of STAND NTPases are the NB-ARC family (nucleotide-binding domain found in human APAF-1, certain plant R proteins, and in *C. elegans* CED-4) [102], and the NACHT family NTPases, named after the four founding STAND members (NAIP, CIITA, HET-E, and TP1) [103]. The NB-ARC and NACHT domains are collectively referred to as the nucleotide-binding and oligomerization domain (or NOD), and proteins that contain these domains are commonly termed NOD-like receptors (NLRs) [104–106] (Fig. 3).

In the classical tripartite STAND architecture, the NOD module is spanned by an N-terminal signaling (or accessory) domain and superstructure-forming pseudo-repeats (20-40-amino acid length) in the C-terminal region [100]. These repeats are proposed to regulate activation of the STAND sensors in response to interactions with diverse types of molecules [107]. Leucine-rich repeats (LRRs) are among the most frequently identified sequences in such architectures and are characteristic of the NBs-LRR family (nucleotide-binding site with leucine-rich repeats), controlling immune cell death in animals and plants [108–113]. NBs-LRRs are also frequently referred to as NLRs (the same acronym as for NOD-like receptors) [104–106]. However, NOD-like receptors more broadly designate immunity-related STAND proteins that exhibit more diverse architectures than the classical NBs-LRR receptors [114, 115]. In prokaryotes, for example, such NLR proteins play immune roles by protecting against phages [115, 116]. These microbial NLRs are proposed to induce RCD in infected bacterial cells to hinder phage propagation – a process termed *abortive infection* [115]. Gao *et al*. have demonstrated that some bacterial and archaeal STANDs – named *Avs* for anti-viral STANDs – can directly recognize hallmark phage proteins and share signaling mechanisms similar to those of their homologs in metazoans and plants [116].

### NOD-like receptors in fungi: from molecular diversity to roles in heterokaryon incompatibility

Fungal genomes encode an abundant and highly diverse repertoire of NOD-like receptors, many of which carry WD40 repeats similar to those of APAF-1 [94, 114, 117]. Ankyrin (ANK) and tetratricopeptide repeats (TPRs) frequently substitute for WD40 repeats in a variety of fungal NLR-like proteins [114]. However, no leucine-rich repeats were identified, and the classical NOD-like architecture of NBs-LRR – prevalent in plants and animals – appeared absent from the analyzed fungal genomes [88]. The fungal NLR-like proteins (hereafter, simply referred to as NLRs) exhibit greater architectural diversity than homologs from other eukaryotic taxa, with more than 14 distinct types of N-terminal signaling modules [94]. Among this diversity, approximately 20% of the fungal NLRs possess a central NB-ARC NOD domain, while most of the remaining receptors carry a central NACHT domain [114]. Based on the type of NOD domain, fungal NLRs therefore resemble those of animals—where NACHT domains predominate—more than those of plants, in which NB-ARC domains are more common [114]. Beyond their structural diversity, the distribution of NLR genes varies across fungal lineages: they are generally abundant in ascomycete and basidiomycete species but absent from yeast genomes, including *Saccharomyces cerevisiae* [114].

Several fungal NLRs have been experimentally characterized in the context of heterokaryon incompatibility, and the family is proposed to play a broader role in fungal immunity and immune cell death [88, 118] (Table 1). NLR genes have been found to determine the outcome of somatic cell fusions between conspecific strains in *N. crassa* [119], *A. fumigatus* [80], *C. parasitica* [69], and *P. anserina* [119–122]. In the latter, two paralogs of the HNWD family (HET-NACHT-WD40 domain organization), reminiscent of the APAF-1 architecture, trigger RCD when co-expressed with incompatible allelic variants of an endogenous glycolipid transfer protein (GLTP) known as HET-C [123, 124] (Fig. 3). The cell death process initiated by the incompatibility between certain HNWD proteins (HET-E and HET-D) and HET-C (GLTP) depends on polymorphisms in the WD40 repeats of the fungal NLRs [121, 125, 126] (Fig. 3). This has led to a model proposing direct binding between the WD40 sensory domain and the HET-C GLTP [127]. The interaction appears analogous to the one described between the WD40 repeats of APAF-1 and cytochrome *c* [128]. Nevertheless, HI-related RCD – and specifically NLR-controlled cell death in this context – is generally lytic and thus fundamentally distinct from the apoptotic programs described in animals.

**Table 1:** Summary of experimental evidence for fungal RCD proteins or domains drawing evolutionary parallels between immune systems.

	In vivo	In vitro	Structure
NLRs	• *N. crassa*: PLP-1 NLR controls germling-regulated death (GRD). • PLP-1 is shown to oligomerize in vivo during cell death. • Conserved HI function in *P. anserina* (HET-Z1 NLR) and *Botrytis cinerea*. • *P. anserina*: HET-E, HET-D, HET-R and HET-Z1. Four NLRs controlling cell death during HI. NWD2 NLR shown to control the HeLo-domain pore-forming protein HET-S, in a ligand-dependent manner. • Mutational analyses for p-loop-dependence of activity for both PLP-1 and NWD2 NLRs. The p-loop motif binds ATP and is conserved on NLR proteins throughout the tree of life.	ND	ND
HeLo/HELL domain proteins	*P. anserina* HET-S HeLo domain is essential for cell death and shown to target the plasma membrane. The domain is cytotoxic in bacteria and yeast. HeLo-like domains (HELL) have similar properties. Experimental work has confirmed the results from *P. anserin*a with a HELL domain from *C. globosum*. Mutational analyses have demonstrated the importance of an extreme N-terminal helix for the membrane targeting and RCD.	HeLo of HET-S binds and permeabilizes liposomes. HET-S oligomerizes in membrane-like environment.	PDB: 2WVO
Signaling amyloids	HET-s(218–289) forms amyloids in *P. anserina* controlling allorecognition RCD. Several amyloid-based RCD systems, involving fungal NLRs have been explored from *P. anserina* and other filamentous fungal species.	Amyloid fibers formation and characterization.	PDB: 2RNM, 6EKA
HET/TIR domain proteins	HET (from heterokaryon) domain proteins are identified on many HI systems in fungi. The domain ( ~ 220-240 aa) has been shown as sufficient to induce cell death when overexpressed. *N. crassa*: TOL, PIN-c, HET-6, HET-e control allorecognition RCD. *P. anserina*: HET-E, HET-D, HET-R, and HET-B all carry a HET domain.	ND	ND
fGSDMs	fGSDMs control cell death in both *N. crassa* and *P. anserina*. *N. crassa*: RCD-1-1, RCD-1-2 target the plasma membrane and oligomerize. Induce cell death in bacteria, yeast and human 293 T cells. *P. anserina*: HET-Q1 fGSDM activated by proteolytic cleavage. Induces cell death in all tested heterologous systems.	Lipid-binding, oligomerization and pore-formation.	PDB: 8JYY,8JYZ

*NLR* NOD-like receptors, *fGSDM* fungal gasdermin, *TIR* Toll-interleukin-1 receptor domain, *RCD* regulated cell death, *ND* Not Determined, *PDB* Protein Data Bank.

Two of the most comprehensively characterized fungal NLRs are the HI-controlling gene *plp-1* from *N. crassa* [119] and the HI-associated gene *nwd2* from *P. anserina* [129, 130]. PLP-1 (patatin-like phospholipase-1) contains an NB-ARC domain with TPR repeats and an N-terminal patatin phospholipase domain. The PLP-1 NLR triggers cell death in germinating asexual spores and hyphae of *N. crassa* in the presence of an incompatible variant of the SEC-9 protein, which is involved in vesicle transport (t-SNARE). Heller et al. have shown that the two proteins interact physically, and that RCD depends on the activity of the patatin domain and the ATPase-binding motif of the NB-ARC domain [119]. PLP-1 also interacts with itself during the cell death process, suggesting that the protein oligomerizes in a manner similar to characterized NLRs from metazoans, plants, and bacteria. A striking observation about the PLP-1/SEC-9 HI system is its apparent independent emergence in multiple fungal lineages, including *P. anserina* [119], *C. parasitica* [69], and *B. cinerea* [131]. An explanation for the apparent convergent evolution has been proposed, suggesting that PLP-1 and SEC-9 function as partners outside the context of allorecognition, and that the NLR might be surveilling the state (or presence) of the t-SNARE protein. This model is inspired by the ‘guard’ strategy employed by some plant NLRs to provide immune defense against pathogens [132, 133]. However, in fungi, these genes accumulate polymorphisms in strains that evolve independently, potentially generating incompatible PLP-1/SEC-9 combinations when such strains undergo somatic cell fusion. The ‘guard hypothesis’ has also been invoked to explain other NLR-based HI systems in *P. anserina* [88].

**Fig. 4:**
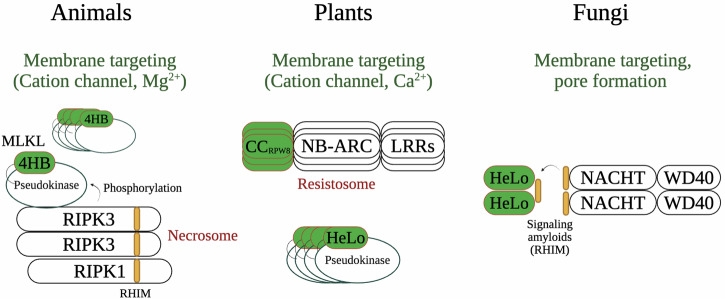
Necroptotic-like cell death operates in three major eukaryotic kingdoms. In mammals, necroptosis depends upon the formation of a protein complex termed the necrosome, formed by the RIP1 and RIP3 kinases. The RIP homotypic interaction motif (RHIM) is a short amyloidogenic sequence that mediates necrosome formation. Activated RIPK3 in the necrosome phosphorylates the pseudokinase domain of MLKL, the executioner of necroptosis. Phosphorylated MLKL aggregates and punctures the plasma membrane, forming a transmembrane cation channel, ultimately inducing cell death. The four-helical bundle (4HB) domain plays an essential role in MLKL membrane targeting. An octameric MLKL complex, composed of two tetramers, opens a cation channel to induce necroptotic cell death. The membrane-targeting module has been characterized in immunity-related cell death processes in plants and fungi. In plants, 4HB homologs can be found in some NLRs and in recently characterized plant MLKL-like proteins. These plant and fungal homologs carry distinct names – RPW8 (Resistant to Powdery Mildew) coiled-coil domain (CC_RPW8_) and HeLo (found in HET-s/LopB) domain. Plant NLRs carrying the CC_RPW8_ play an important role in the plant immune system, controlling localized necrotic cell death in response to pathogens, termed the *hypersensitive response* (HR). Some such plant NLRs have been shown to form a pentameric complex named the resistosome to induce HR. The HeLo domain has also been experimentally characterized in fungi. Some fungal NLRs control HeLo-domain cell death effectors through signal transduction involving amyloid-forming domains, some of which appear homologous to mammalian RHIM. HeLo (and CC_RPW8_) rely on an extreme N-terminal α-helix to attack the plasma membrane. While fungal HeLo domains are shown to act as pore-forming proteins in vitro, it is currently unknown if they form cation channels.

NWD2 (NACHT-WD40s) is a member of the *nwd* family of NLRs in *P. anserina* [125], whose reference genome encodes 77 different NLR genes (Fig. 3) [114]. Three different *nwd* genes encode allorecognition determinants (*het-e*, *het-d*, and *het-r*), while *nwd2* is involved only indirectly in an HI system (reviewed below) [134]. The discovery and characterization of *nwd2* have established that some fungal RCD pathways are related to *necroptosis* [20] and to plant cell death programs like the *hypersensitive response* [11, 135](Fig. 4). These findings have greatly contributed to the integration of HI and fungal RCD into the field of comparative immunology [97, 98].

## Evolutionary links between fungal cell death, mammalian *necroptosis*, and the *hypersensitive response* in plants

*Necroptosis* is involved in the pathophysiology of a range of human diseases [136] and is considered an important anti-viral immune cell death [137]. Necroptotic cell death can be triggered by a variety of cell surface receptors – such as TNFR (tumor necrosis factor receptor) or certain Toll-like receptors (e.g., TLR4) [138] – as well as by intracellular sensors of nucleic acids [139–141]. The necroptotic pathway is typically engaged when apoptosis and the pro-apoptotic caspase-8 are inhibited, in some cases directly by the invading pathogen [142]. The initiation of necroptosis requires the formation of a large multimeric heterocomplex of the kinases RIPK1 (receptor-interacting protein kinase 1) and RIPK3, known as the *necrosome* [143, 144]. Oligomerization of the necrosome is mediated by an 18–22 amino acid-long RIP homotypic interaction motif (RHIM), conserved in both RIPK1 and RIPK3 [145–147]. Once recruited into the necrosome, RIPK3 phosphorylates the mixed-lineage kinase-like (MLKL) protein, which then oligomerizes to form a cation channel in the plasma membrane, culminating in necroptotic cell death [96, 148, 149] (Fig. 4). MLKL functions as a cell death executioner, and its involvement in RCD is a hallmark of necroptosis.

In the following paragraphs, we review the evolutionary links between necroptosis and recently described fungal RCD processes.

### A subset of fungal NLRs use amyloid signaling to activate cell death effectors

The RHIM domain, or motif, which is essential for the formation of the necrosome, forms a functional *amyloid* [145]. Amyloids are highly ordered, strictly cooperative, β-sheet-rich protein structures often associated with neurodegenerative diseases [150, 151]. Many amyloid aggregates are the result of protein misfolding. Functional amyloids, however, are integral to various biological processes and are shaped by evolutionary forces [152–154].

In fungi, amyloid domains have been integrated into RCD pathways as signal-transducing modules between a NOD-like receptor and a downstream signaling partner [155]. In general, the two or three genes that constitute the functional unit are genomically clustered [129]. The first such gene cluster described, identified in *P. anserina*, comprises the *nwd2* NLR-encoding gene, and *het-S*, which encodes a pore-forming protein [129, 130]. The short amyloid signal-transducing sequence, consisting of 21 amino acids and termed R0 (repeat zero), is situated at the extreme N-terminus of NWD2 [129, 130]. HET-S carries two C-terminal amyloid pseudo-repeats – R1 and R2 – and an N-terminal HeLo domain, which targets the plasma membrane when the protein is activated by the NWD2 NLR [156–158]. The three amyloid-forming repeats (R0, R1, and R2) exhibit strong sequence similarity and appear to adopt the same amyloid fold [130]. A model for the signaling mechanism proposes that activated NWD2 oligomerizes, bringing the R0 motifs into proximity within the NLR protein complex, which in turn promotes the cooperative folding and the emergence of the amyloid fold. The newly formed amyloid then serves as a structural template for the R1-R2 pseudo-repeats of the HET-S cell death effector [129, 130, 159]. The transconformation of HET-S C-terminal amyloid domain triggers the activity of the cytotoxic HeLo domain [159]. The discovery of NLR-based amyloid signaling, along with insights into its molecular mechanism, has been advanced through studies of a naturally occurring cytotoxic-dead HET-S variant, known as HET-s (small s) [134]. HET-s is a model prion protein extensively used to investigate the fundamental properties of amyloid folds [160].

### Natural diversity of fungal signaling amyloids includes RHIM-like motifs

A high-resolution SSNMR structure of the HET-S/s amyloid domain has been reported, revealing a left-handed β-solenoid (β helix) fold [161]. The triangular, highly hydrophobic amyloid core of HET-S/s bears slight similarity to the N-shaped amyloid core of RIPK3 RHIM homotypic oligomers [162]. An evolutionary relationship between RHIMs and the HET-S R1-R2 amyloid core-forming repeats has previously been proposed based on molecular modeling [163]. NLR-dependent signaling amyloids in fungi have been undergone extensive diversification [155]. Among the diverse amyloidogenic sequences, one widespread signaling amyloid – named pseudo-palindromic, or ‘PP’ – appears to share significant sequence similarity with RHIM [129]. The PP (fungal RHIM) domain was first characterized as a functional signaling amyloid using a three-gene cluster from *Chaetomium globosum* [95], and more recently, a two-gene cluster from *P. anserina* [164]. In the latter case, Bardin et al. explored HELLP (HeLo-like PP) and PNT1 (named after the NLR architecture PP-NB-ARC-TPR), encoded by a two-gene cluster analogous to *het-S*/*nwd2*. The authors show that *P. anserina* PP/RHIM domain can ‘cross-seed’ – that is, signal-transduce between two distinct amyloids – in vivo with human RIPK1 and RIPK3 RHIMs [164]. PP/RHIM signaling has also been reported in bacteria, where, similarly to fungi, a much greater diversity of signaling amyloids has been uncovered compared to metazoans [165]. The increased diversity of the signaling amyloids likely contributes to higher specificity in the signaling process, as suggested by experimental data from the HET-S-related amyloid motifs (HRAM) subfamily [166, 167].

#### Evolutionary links between fungal HeLo domains and the membrane-targeting mammalian protein MLKL

The necrosome is activated by phosphorylation of the MLKL protein, the cell death executioner of necroptosis [168–170]. Phosphorylation by RIPK3 of a regulatory loop in the pseudokinase domain of MLKL unleashes its N-terminal, membrane-targeting four-helix bundle (4HB) domain [171, 172]. Phosphorylated MLKL oligomerizes and localizes to the cell periphery, where the 4HB domain perforates the plasma membrane to form ion channels [173–175]. Remarkably, an evolutionary link has been established between the 4HB domain of MLKL and the HeLo and HeLo-like (HELL) domains of HET-S and HELLP, respectively [95]. Several other fungal domain annotations have been grouped with the membrane-targeting HeLo and HELL in the superfamily of 4HB MLKL homologs [94]. Using AlphaFold molecular modeling, Wojciechowski *et al*. have established that the helical folds of the HeLo and HeLo-like domains present the highest similarity with MLKL 4HB [94]. The main difference between the generated models and the structure of 4HB domain lies in the variable lengths of the helix located at the extreme N-terminus (helix 1) in some molecular models [94]. The N-terminal helix of the HET-S HeLo domain has been shown to play a key role in membrane targeting [158] and it is hypothesized that a similar mechanism of membrane disruption and pore formation is conserved for the other members of the protein family [94].

The fungal HeLo and HeLo-like domains have also been conserved in plants [94, 95]. In plants, HeLo homologs occur as N-terminal domains on a subset of NLRs [176]. Recently, the structure of one such NLR – ZAR1 (HOPZ-ACTIVATED RESISTANCE 1) – has been solved with cryo-EM, revealing the formation of a pentameric signaling hub, termed a ‘resistosome’ [177, 178]. The HeLo/4HB homologous domains of ZAR1 molecules cluster atop of the doughnut-shaped resistosome, forming a calcium-permeable channel [179]. Noteworthy, a large number of fungal NLRs present similar protein architectures, where HeLo, HeLo-like, SesA or Goodbye domains are directly integrated at the N-terminus of the molecular receptors [88, 94, 114]. Thus, in fungi, some NLRs use signaling amyloids like RHIM to activate downstream effector or cell death-inducing domains, while in other cases, these domains are integrated to the receptor in an alternate ‘all-in-one’ architecture. Intriguingly, plants also possess in their immune arsenal bona fide MLKL homologs [180]. Future work should be focused on exploring more broadly the HeLo/4HB proteins in fungi and clarify the extent of conservation between necroptosis and fungal necroptotic-like cell death.

## Microbial gasdermins draw evolutionary parallels with mammalian *pyroptosis*

**Fig. 5:**
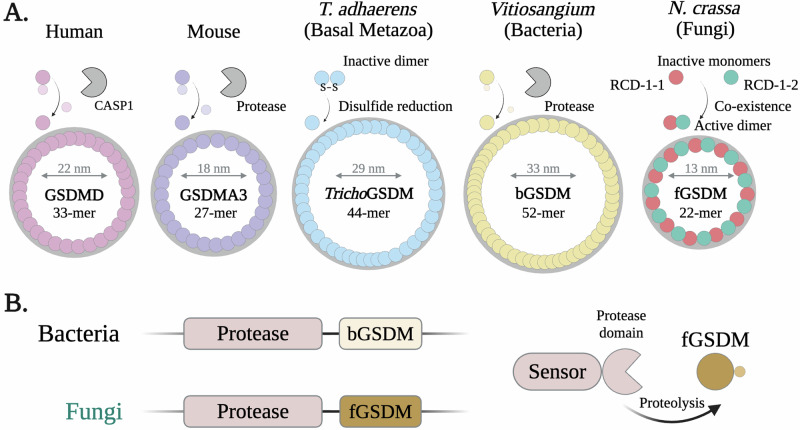
Gasdermins are a transkingdom family of pore-forming proteins controlling immune cell death. **A** Shown are cartoon representations of gasdermin pores with known structures. The two allelic variants RCD-1-1 and RCD-1-2 from *N. crassa* are currently the only known gasdermins to form heteromeric pores, composed of eleven alternating RCD-1-1/RCD-1-2 dimers. The RCD-1 fGSDM is not controlled by proteolytic cleavage, like most other gasdermins, but by the coexistance in the same cell of the two allelic variants. This coexistance occurs only when *N. crassa* strains from the *rcd-1-1* and *rcd-1-2* antagonistic genotypes undergo cellular fusion. **B** The majority of gasdermins in microorganisms (bacteria and fungi) are genomically clustered with protease-encoding genes. These multidomain proteases are putative molecular sensors that control the adjecently encoded gasdermin. PDB IDs: GSDMD (6VFE), GSDMA3 (6CB8), TrichoGSDM (8JYW), bGSDM (8SL0), fGSDM (8JYZ).

Recent findings indicate that the fungal RCD machinery can rely on cell death programs resembling mammalian *pyroptosis* [181, 182]. Like necroptosis, pyroptosis is a highly inflammatory and lytic cell death in mammals, with diverse immune and physiological roles [22, 183, 184]. The pyroptotic cell death pathway relies on a family of pore-forming proteins, known as *gasdermins*, which act as executioners of cell death [185, 186]. The gasdermins are regulated by proteolytic cleavage, exerted by different caspases and some serine proteases, depending on the specific gasdermin [187]. In humans, the gasdermin family comprises six members (GSDMA-GSDME and Pejvakin) with GSDMD being one of the first and best-characterized gasdermin proteins [188, 189]. GSDMD is specifically cleaved by pro-inflammatory caspases (CASP1, CASP4, and CASP5), with CASP1 acting notably downstream of various NLR-based inflammasomes [189]. The caspase removes the inhibitory C-terminal domain of GSDMD and liberates the N-terminal pore-forming domain, which then binds to the plasma membrane and oligomerizes into highly ordered pores [190–192] (Fig. 5). Gasdermin homologs have been identified in species across the metazoan branch, including in some basal clades, and in both fungi and bacteria [92, 193]. Remarkably, these distant microbial gasdermins have retained the immune-related role – as determinants of allorecognition in fungi, and as components of anti-phage defense systems in bacteria [92, 193].

### Fungal gasdermins (fGSDMs) in allorecognition: cell death mechanisms and structural insights

**Fig. 6:**
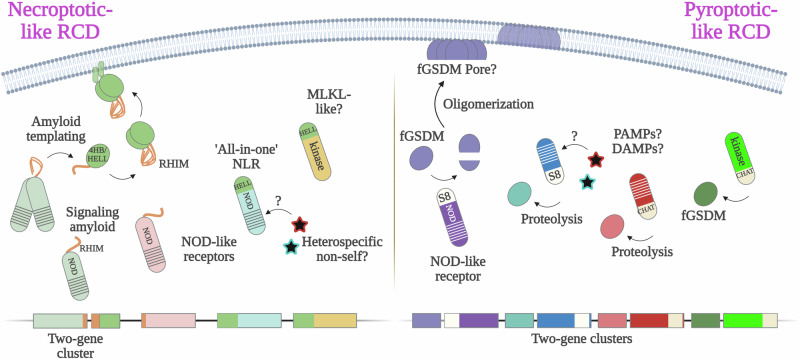
Necroptotic and pyroptotic pathways draw several evolutionary parallels between fungal and metazoan RCD machineries. Fungal genomes encode a variety of putative molecular sensors controlling pore-forming proteins, such as fGSDMs. Some of these sensors belong to the NOD-like receptor (NLR) family. A subset of NLRs use signaling amyloids – some of which appear related to the metazoan necroptosis-controlling RHIM (RIP homotypic interaction motif) – to activate downstream membrane-targeting proteins. The latter are characterized by a helical bundle domain, named HeLo-like (HELL) or HeLo, evolutionary related to the four-helix bundle (4HB) of the necroptosis executioner protein MLKL (Mixed lineage kinase domain-like pseudokinase). The signaling mechanism is based on ‘amyloid templating’ or the transmission of structural information from the NLR-based signalosome to the RCD executioner protein. Certain NLR proteins carry a HeLo/HELL domain at their N-terminal end. This ‘all-in-one’ architecture is analogous to some plant NLRs, which carry a coiled-coil (CC) domain as an N-terminal effector/signaling module. Remarkably, some HELL domains are integral to proteins with kinase/pseudokinase domain, reminiscent of the original MLKL architecture. Similar diversity of molecular receptors has been uncovered in the fungal pyroptotic-like pathways. A subset of the fGSDM-controlling proteins are NLR sensors, carrying an S8 serine protease or a CHAT caspase-like domain. The protease domains can frequently be directly fused to superstructure-forming repeats (WD40, TPR or ANK) or other predicted kinase-like domains. Notably, the fungal necroptotic-like and pyroptotic-like RCD pathways are often encoded in two- or three-gene clusters, sharing thus some characteristics with prokaryotes. The number of such clusters can vary widely between species. A major open question regarding these RCD pathways in fungi is related to the nature of stimuli that activate the identified diverse putative sensors.

Gasdermin-like genes are abundant in fungal genomes and have been uncovered through studies of heterokaryon incompatibility (HI) and allorecognition systems [92, 193]. Fungal gasdermin (fGSDM)-based allorecognition systems have been described in at least two different ascomycete species, *Podospora anserina* and *Neurospora crassa* [181, 182, 194]. These two fGSDM-based HI systems – like HI systems in general – are proposed to have emerged from broader signaling pathways and to have been evolutionary selected for their prophylactic cell death function [97, 98]. Under such a model, the *het-Q1*/*het-Q2* system in *P. anserina* and the *rcd-1-1*/*rcd-1-2* in *N. crassa* likely derive from pre-existing fGSDM-mediated cell death pathways (Fig. 6). This would explain the striking differences between the two HI systems; notably, in *N. crassa*, the allorecognition cell death is triggered by the co-expression of two divergent gasdermin alleles (*rcd-1-1* and *rcd-1-2*) within the same cell, whereas the HI system in *P. anserina* involves a gasdermin-encoding gene (*het-Q1*) and a serine protease-encoding gene (*het-Q2*) [182, 194]. The *het-Q1* and *het-Q2* genes are idiomorphs (different genes located at the same locus), and genomic rearrangements have also been reported for the *rcd-1* locus [181, 194]. The two fGSDM-based incompatibility systems have recently been reviewed in greater depth elsewhere [193].

A notable difference between fungal and metazoan gasdermins lies in the length of their inhibitory C-terminal domains, where no sequence or structural similarity has been identified between the two clades [182, 194]. In metazoans, the inhibitory domain is typically of similar length to the pore-forming domain, which itself shares some sequence homology and structural similarity with the cytotoxic N-terminal domain of fGSDMs. In contrast, microbial gasdermins possess a much shorter inhibitory domain relative to their membrane-targeting cytotoxic domain [194]. For example, the proteolytically processed HET-Q1 gasdermin loses a ~ 5-kDa fragment from its C-terminus, likely cleaved at residue F238 by the subtilisin-like serine protease HET-Q2 [194]. Such short inhibitory domains have been described in bacterial gasdermins, whose inhibition mechanisms differ significantly from those of mammalian gasdermins [195].

Several functional similarities have been identified between fungal and metazoan gasdermins, including oligomer formation, lipid recognition profiles, and regulation by proteolysis [182, 194]. In vivo, RCD-1-1 (257 aa) and RCD-1-2 (244 aa) show plasma membrane affinity, which depends on a patch of positively charged residues situated on a pair of amphipathic α-helices [182]. Similar structural features of functional importance are conserved in human GSDMD and murine GSDMA3 [190, 196]. When co-expressed heterologously, the two RCD-1 allelic variants physically interact to trigger pyroptotic-like cell death in human 293 T cells, underscoring the autonomy of the allorecognition system [182]. Li *et al*. have recently reported the crystal structures of both monomeric RCD-1 variants, which share nearly identical protein folds [197]. The structures the two gasdermins most closely resemble those of mammalian gasdermins. Remarkably, the authors have also solved a Cryo-EM structure of heteromeric transmembrane pore formed by alternating RCD-1-1 and RCD-1-2 subunits (Fig. 5). The uncovered mechanism of pore-formation relies on the heterodimerization of the two allelic variants in a lipid environment, before shaping the observed 11-fold pore complex [197, 198]. This mechanism provides a molecular explanation for the allorecognition cell death process, as the two allelic variants coexist only after somatic fusion of a strain from the *rcd-1-1* genotype with a strain from the *rcd-1-2* genotype.

#### Protease-clustered fGSDM genes and the ancient origins of pyroptotic-like cell death

Most fGSDMs appear to be regulated by proteolytic cleavage, and it has been reported that more than 80% of fGSDM genes are genomically clustered with genes encoding a protease domain [194]. The encoded proteases represent multidomain molecular sensors, with a large fraction of the proteins carrying super-structure-forming motifs (WD40, ANK, and TPR) next to the protease domain. A small subset of the molecular sensors consists of NLR proteins with the protease domain as the N-terminal signaling module [194]. The majority of protease domains have been classified as serine proteases and approximately 17% as cysteine proteases, among which is the caspase-like CHAT domain (Caspase HetF Associated with TPRs) [199]. CHAT proteases belong to the same clade as mammalian caspases and appear to adopt folds showing a high structural similarity to the separase family of cysteine proteases [199, 200]. The functional relation between the fGSDMs and the protease-carrying receptors has been explored within a two-gene cluster from *P. anserina*, demonstrating the activation of the gasdermin by a TPR-bearing CHAT protease [194]. Similar genomic clustering of gasdermin genes and protease-encoding genes has been reported in bacterial and archaeal genomes [195, 201]. These findings suggest that pyroptotic-like cell death has an extremely ancient evolutionary origin and that fGSDMs, considering their abundance and distribution, are important actors in the fungal cell death machinery. Future research will be needed to uncover the regulatory mechanisms controlling the protease-bearing proteins that activate fGSDMs, and to explore the roles of these two-gene clusters in other biological processes such as secretion.

## Comparative immunology in Fungi: the end of the beginning

In summary, it is now clear that fungal genomes harbor a variety of genes with evolutionary ties to molecular players integral to all three core mammalian immune-related RCD pathways: *apoptosis*, *necroptosis* and *pyroptosis*. Most of these evolutionary parallels extend from bacteria to metazoans, highlighting the trans-kingdom history of the cell death machinery. This conservation encompasses all aspects of regulated cell death, including execution, signal transduction and signal perception. Considering the latter, the NOD-like receptor family emerges as a significant thread in the evolutionary history of immune sensing. Fungal NLR proteins participate in both necroptotic- and pyroptotic-like regulated cell death, and in some cases, they display protein architectures similar to those of the pro-apoptotic mammalian NLR-like protein APAF-1. In addition, several signaling N-terminal domains of fungal NLRs share ancestry with immune signaling modules in other taxa, including plants and bacteria. Two notable examples are the HET domain, proposed as a distant relative to the Toll-like receptor (TIR) domain [83, 114], and members of the purine nucleoside phosphorylases and uridine phosphorylases (PNP_UDP) family [94]. Both of these signaling modules exhibit hydrolase activity (to NAD^+^ and ATP, respectively) and have been shown to function in immune-related cell death in different eukaryotic kingdoms as well as bacteria [202–206]. These findings further extend the evolutionary ties between eukaryotic and prokaryotic RCD genes, while also expanding the fungal immune arsenal.

At present, as the outlines of the fungal immune-related gene families become clearer, many questions remain regarding the description and conceptualization of the fungal immune system. In the first instance, while some evolutionary links have been unveiled between fungal RCD and the core innate immune pathways in metazoans (necroptosis and pyroptosis), the cellular characterization of the fungal cell death pathways remains incomplete, complicating comparisons, analogies, and the adoption of a common vocabulary within the Opisthokonts. A better understanding of the RCD manifestations at the cellular level, triggered by different molecular receptors, would be needed to eventually delineate distinct cell death pathways. Most of the presently characterized cell death processes, especially in the context of heterokaryon incompatibility, appear to be lytic (leakage of cytoplasm), drawing parallels with *necroptosis*, *pyroptosis*, and *PANoptosis*. However, it is unclear if specific hallmarks can be associated with different allorecognition systems and inputs from distinct molecular sensors or following the activation of specific cell death–inducing proteins, allowing us to identify separate RCD programs.

In the perspective of comparing the innate immune mechanisms of distant organisms, we would need to decipher the ones operating in fungi in much greater details, including their regulation, roles, and integration with other fundamental physiological processes. Novel experimental model systems, recently developed to study fungal-bacterial interactions [207], may help uncover more specifics about fungal RCD in response to heterospecific non-self. Despite our limited understanding of the fungal immune system, it appears to display features of both eukaryotic and prokaryotic lineages. The observed genomic clustering in many fungal genomes of sensors and cell death executioner genes resembles the numerous bacterial anti-phage operons clustered together in *defense islands* [208]. It is conceivable that microorganisms, independently of their eukaryotic or prokaryotic classification, share certain immunity-related adaptations setting them apart from differentiated higher eukaryotes (i.e. plants, animals), due to morphological similarities (limited differentiation of somatic cells), common ecological niches, and similar lifestyles.

As evidence accumulate for the prokaryotic origin of the molecular RCD machinery [209, 210], and its role in the emergence of multicellularity appears highly plausible [211, 212], fungi occupy a particular space on the multicellularity spectra with their syncytial – sharing cytoplasm and nuclei between cells – filamentous networks as organismal body structure. Besides the syncytial aspect of fungi, their ability to undergo regular vegetative anastomosis, or cellular fusions between distinct fungal colonies, adds some taxon-level idiosyncrasy to the notion of ‘individuality’, which is of paramount importance in immunology [213]. The extent to which cellular organization and different lifestyles shape the evolution and mechanisms of immune cell death and broadly of immunity [214], are particularly intriguing questions to explore in the framework of trans-kingdom comparative immunology. Finally, elucidating the molecular mechanisms of fungal RCD could open the door to designing new strategies and exploiting these endogenous cell death programs to thwart some pathogenic fungal species, of major importance for human health, agriculture and food security [215, 216].
